# Association between endometriosis and hyperprolactinemia in infertile women

**Published:** 2015-03

**Authors:** Seddigheh Esmaeilzadeh, Parvaneh Mirabi, Zahra Basirat, Mahtab Zeinalzadeh, Soraya Khafri

**Affiliations:** 1 *Fatemeh-zahra Infertility and Reproductive Health Research Center, Babol University of Medical Sciences, Babol, Iran.*; 2 *Department of Biostatistics and Epidemiology, Faculty of Medicine, Babol University of Medical Sciences, Babol, Iran.*

**Keywords:** *Endometriosis*, *Infertility*, *Hyperprolactinemia*, *Laparoscopy*

## Abstract

**Background:**

The association of endometriosis with hyperprolactinemia is controversial.

**Objective:**

The present study aimed to determine the frequency of endometriosis and association of prolactin with endometriosis in infertile women.

**Materials and Methods:**

256 infertile women who underwent diagnostic laparoscopy for the evaluation of infertility, referred to Fatemezahra Infertility and Reproductive Health Research Center were included in a cross-sectional study. The presence of endometriosis was evaluated. To investigate the association of endometriosis with hyperprolactinemia, the patients whose infertility was not caused by endometriosis were included as control group. Serum prolactin (PRL) level was measured in both groups. The comparison of basal serum PRL levels between the two groups was performed, using independent t-test. One way ANOVA was used to determine PRL association with endometriosis stages.

**Results:**

The frequency of endometriosis was found to be 29%. PRL levels were significantly higher in endometriosis group compared to control group (23.02±1.25 vs. 17.22±1.22 respectively, p=0.004). Statistically significant associations were found between staging of endometriosis and prolactin levels (p=0.01).

**Conclusion:**

Hyperprolactinemia may be associated with endometriosis and its progression.

## Introduction

Endometriosis is the most common benign disease affecting approximately 10-15% of reproductive age women and frequently (30±60%) is associated with infertility. It is defined as the presence of endometrial tissue outside the normal uterine cavity (1). Symptoms include dysmenorrhea, dyspareunia, subfertility, dyschezia, and pelvic or lower abdominal pain, abnormal bleeding and chronic fatigue (2).

Despite the wide associated morbidity and health care costs, the true prevalence, incidence and risk factors of endometriosis remain unknown and it is difficult to quantify since very wide ranges have been reported in the literature (3, 4).Since laparoscopy is the only reliable and standard investigation for diagnosis and generally is not performed on women without symptoms or physical finding that strongly suggest the disease (4). In women with pelvic pain, the prevalence of endometriosis ranges from 12-32% and 25-50% of infertile women have endometriosis that 30-50% of women with endometriosis is infertile. The overall prevalence of endometriosis in women undergoing a laparoscopy for the evaluation of infertility is 9-50%. The incidence of endometriosis seems to be increasing (3, -).

Endometriosis can be divided into four stages of severity (stage I= minimal disease; stage IV= severe disease) as defined by the American Society of Reproductive Medicine (ASRM) (8). It impaired women's health related quality of life, impacting on their activities, sexual and non-sexual relationships, and fertility (9). The pathophysiology of endometriosis and pathogenesis are not clear and endometriosis associated idiopathic infertility is still among the most difficult problems facing the gynecologist    (10) . Endometriotic implants may also secrete prolactin (PRL) and possibly cause ovarian dysfunction. Researchers have since tried to establish this relationship.

The main conclusions of some studies indicate an increased number of activated macrophages and their immune mediators, which may substantially interfere with reproduction at various levels, including gamete function, fertilization, embryo development, and embryo implantation. Furthermore, women with endometriosis have reduced cellular immunity and more specifically a decreased natural killer activity in peripheral blood and in peritoneal fluid (11). It is also well known that parameters associated with stress can significantly alter several immunological parameters, consisting of the number of cells as well as their function. PRL is discharged in response to stress or stimuli, although its true role in response to stress is not known. As both endometriosis and hyperprolactinemia are associated with infertility it became an attractive theory to implicate raised PRL levels as the cause for infertility in women with endometriosis (12, 13).

An elevated level of PRL produces anovulation because it prevents LH pulsatility and interferes with hypothalamic function through the blockage of estrogen receptors. Actions on the ovary maybe due to a decreased affinity of LH receptors in the corpus luteum and an associated decrease in the production and secretion of progesterone, resulting in not only anovulation but also suppression of follicular maturation and an inadequate corpus luteum, often termed as “short luteal phase.” These have all been found to exist in infertile women with endometriosis of varying degrees (13). 

There have been several studies in the literature measuring basal serum PRL levels in infertile patients with or without endometriosis. Some studies reported hyperprolactinemia as a possible cause of infertility, whereas the remaining studies found no statistically significant elevation of serum PRL concentration between the two groups (12-15). The true mechanisms of action of hyperprolactinemia in patients with endometriosis associated infertility have not been perfectly reported, therefore studies only suggest the relationship between endometriosis and abnormal PRL secretion which in turn are limited in number and their results are controversial. One study reported an elevation of baseline serum PRL in infertile and fertile women with minimal and mild endometriosis as compared with both fertile and infertile patients without endometriosis (16). But another group observed a direct correlation between PRL secretion and disease stage, with serum PRL concentration progressively is increased from stage I-IV (5). There had been several studies that showed no significant relationship between basal PRL levels and the stage of endometriosis (12, 14). 

This evidence gave interest us to determine whether or not the presence of endometriosis and its various stages are related to hyperprolactinemia.

## Materials and methods

This cross-sectional study was conducted at the Fatemezahra Infertility and Reproductive Health Research Center from January 2009 to September 2013. The study was approved by Ethics Committee of Babol University of Medical Sciences. All subjects were given information about the research methodology and entered the project after signing the informed written consent form. To assess the prevalence of endometriosis, medical record of the patients with primary or secondary infertility, subjected to diagnostic laparoscopy and diagnosed to have endometriosis as the cause of infertility and patients without the disease or medicine consumption that cause hyperprolactinemia, were included in the study. 

To investigate the association of endometriosis with hyperprolactinemia, the patients whose infertility was not caused by endometriosis were considered as control group. Inclusion criteria for the control group were as follows: women in the reproductive age undergoing laparoscopy for infertility, without any evidence of endometriosis in the laparoscopy documents, without the presence of the disease or medicine consumption that cause hyperprolactinemia. We excluded the patients with previous endocrine disorders like pituitary adenoma, thyroid disorder, polycystic ovarian syndrome and patients those were using drugs that could affect PRL such as tranquilizers, dopamine-antagonist, and antiemetic drugs.

**Figure 1:**
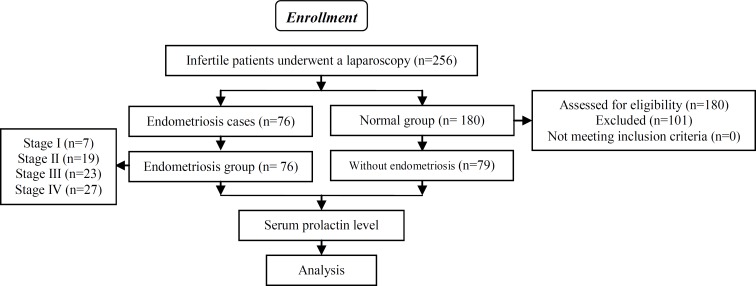
Flow of participants through the study.

The number of samples in the control group was determined based on the number of cases with endometriosis diagnosed in the first stage of the study (Figure 1). The medical records of all subjects were reviewed for demographic data, clinical information and prolactin level. Demographic details were noted e.g. age, education, and body mass index. Extracted clinical data were divided into three categories: 1) Presenting signs and symptoms, duration of infertility, menstrual cycle’s pattern, dysmenorrhea and dyspareunia. 2) Serum prolactin, 3) laparoscopic findings.

In our laboratory serum PRL was measured by Diasorin company kit manufactured by Spain and liazon apparatus and chemo immuno luminance (CLIA) method. We considered PRL secretion as normal if basal serum level was less than 25 ng/ml    (17) . Laparoscopic staging was based on the American Society of Reproductive Medicine (ASRM) scoring for endometriosis which divided the findings into four categories of severity. "1) Stage I (minimal) isolated superficial disease on the peritoneal surface with no significant adhesions. 2) Stage II (mild) scattered superficial disease on the peritoneal surface and ovaries totally less than 5 cm aggregate with no significant associated adhesions. 3) Stage III (moderate) multifocal disease both superficial and invasive that may be associated with adhesions involving the Fallopian tubes and/or ovaries. 4) Stage IV (severe) multi focal disease both superficial and invasive including large ovarian endometriomas, usually associated with adhesions both firmly and dense, involving the Fallopian tubes ovaries and cul-de-sac" (18). 

**Statistical analysis**

The data were entered and analyzed using the statistical package for the social sciences (SPSS software version 19.0 for windows, Chicago, IL). The frequency of endometriosis based on laparoscopic diagnosis was calculated. The comparison of basal serum PRL levels between the two groups (endometriosis group and compare group without endometriosis) was performed, using independent t-test and linear regression. One way-ANOVA was used to determine PRL associated with endometriosis stages. A probability value of less than or equal to 0.05 was considered significant.

## Results

The study included 256 women who were evaluated for infertility. All women underwent laparoscopy and in 76 patients (endometriosis group) endometriosis was diagnosed that include mostly (35.5%) stage IV endometriosis. Others (n= 180) did not have endometriosis. According to the inclusion criteria of the control group 101 patients were excluded. (36 patients had poly cystic ovary, 24 patients had thyroid disorders, 31 women consumed medicines which affect PRL level, 3 patients had pituitary adenoma and 7 patients had missing data in their records) (Figure 1).

Patients with endometriosis had a longer duration of infertility. The total frequency of endometriosis was 29%, including stage I 9.2% (7), stage II 25% (19), stage III 30.3 % (23) and stage IV 35.5% (27) endometriosis. The mean age of patients with endometriosis was 30.79±5.03 years and 28.8±6.07 years for controls. Independent students’ *t*-test showed a significant difference between the two groups. There were no significant differences between the groups in terms of education, body mass index, and primary infertility (Table I). The mean PRL level in the endometriosis group was (23.02±1.25 ng/mL) while in the controls was (17.21±1.22 ng/mL).

The PRL levels were significantly higher in infertile women with endometriosis vs. the infertile women without endometriosis (p=0.004) (Figure 2). Linear regression analysis showed that amongst the variables age, duration of infertility and endometriosis, only age (p=0.03, β=0.38) and endometriosis (p=0.01, β=5.65) had a significant difference with prolactin level. A statistically significant relationship was found between the basal serum PRL level and the stage of endometriosis (16.98±1.29 ng/mL for stages I; 18.07±1.50 ng/mL for stages II; 25.59±1.96 ng/ mL for high stages III-IV) compared to healthy controls (17.21±1.22 ng/mL) (p=0.01).

**Table I:** Description of the study population (n=155)

**Characteristics**		**Endometriosis group (N=76)**	**Control group (N=79)**	**p-value**
Age (y)*		30.80 ± 5.03	28.84 ± 6.10	0.03 a
Body Mass Index (kg/m2)*		25.81 ± 4.70	26.35 ± 4.41	0.55 a
Duration of infertility (y) *		5.29 ± 3.7	3.54 ± 2.54	0.001 a
Education (n)**				0.812 b
	Elementary education	9 (11.8)	12 (15.2)	
	High school	14 (18.4)	13 (16.5)	
	College education	53 (69.7)	54 (68.4)	
Primary infertility (%)		85.5	74.7	0.09 b
Dysmenorrhea(%)		75	64	0.12 b
Dyspareunia(%)		56	25	0.001 b

a : Students' *t*-test

b : ^2^

* Data are presented as mean ± SD.

** Data are presented as n (%)

## Discussion

In the present study, infertile patients with endometriosis were compared to infertile patients without endometriosis; the case group was significantly older vs. the controls (30.8±5 vs. 28.8±6 respectively, p=0.03). According to Speroff the mean age at the time of diagnosis of endometriosis ranges between 25 and 35 years (18). Although, in Pauwel *et al* study there was no consistency compared to our study. In their study, no significant difference was shown in the case and control mean age (19). Our results revealed that the frequency of endometriosis in infertile patients was 29% which is consistent with the findings of other studies (3, 7).

The findings of this study, however, cannot be generalized to the population in large sample size because we investigated only those patients who underwent diagnostic laparoscopy. A significant relationship was found between the scoring of endometriosis and hyperprolactinemia between two groups. Many researchers have investigated the relation between serum PRL levels and endometriosis and further recommend the suppression of PRL levels in these patients as a way of improving their fecundity rate, Although it has been widely accepted that endometriosis can adversely influence fecundity rate and pregnancy, but the results are controversial (20).

However, the mechanism has not been identified. Some studies proposed that the infertility in endometriosis patients mainly depends on the impaired ovarian reserve and reduced ovarian response, as indicated by higher FSH, lower anti-Mullerian hormone not detect a significant increase in PRL levels in women with endometriosis, so ruling out hyperprolactinemia as a cause of infertility (12-14). Gregoriou *et al* found a direct correlation between endometriosis stages, with serum PRL concentration progressively increasing from stage I (minimal) to stage IV (severe), a pattern was also observed in the present study, in which infertile women with moderate stage to severe endometriosis presented significantly higher PRL concentrations compared to control group (15). In a similar study serum PRL levels were significantly higher in infertile women with stage III-IV endometriosis (28.9±2.1 ng/mL) than in healthy controls (13.2±2.1 ng/mL), but they did not detect a significant difference in PRL concentrations in peritoneal fluid or follicular fluid, between case and control groups (5).

The ability of ectopic endometrium to secrete PRL is controversial. While the normal endometrium retains the ability to discharge prolactin in the late luteal phase. Studies on PRL levels in the peritoneal fluid show no evidence that the implants secrete prolactin. Machida *et al* reported no significant relationship between basal PRL levels and the stage of endometriosis after analyzing the samples from 70 patients with and without endometriosis (14). Based on the data presented in some studies, infertile women with endometriosis exhibit latent hyperprolactinemia, which is more evident in infertile women who fail to, become pregnant after several therapeutic schemes. However, more studies aimed at identifying the role of PRL in infertile women with endometriosis are required.

Acien *et al* and He *et al* also found significantly higher basal PRL levels in patients with infertility and endometriosis (20). On the contrary, other investigators however, did not find a significant difference between the groups in terms of basal PRL levels (21, 22). Both the above mentioned studies chose low sample size maybe, that’s why, the results were not significant. Haney *et al* also did not observe a variation of PRL concentration in the peritoneal fluid of women with endometriosis, concluded that the ectopic endometrium of women with endometriosis does not secrete PRL in amounts sufficient to elevate peritoneal fluid concentrations (23). 

On the other hand Hao *et al* observed, not only that the ectopic implants of endometriosis discharge PRL in a significant way, but also that there is a significant association between PRL secretion by ectopic cells and the scoring of endometriosis (24). Although there is a controversy in the literature, we believe that hyperprolactinemia may be associated with endometriosis and its progression, as shown in the present results and in agreement with most of the evidence reported in the literature.

## Conclusion

The preponderance of evidence of the present study suggests that hyperprolactinemia does exist in infertile patients with endometriosis. However; further studies of basal PRL concentrations in various stages of endometriosis are needed to confirm this relationship.
